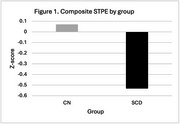# Short‐term practice effects are reduced in Subjective Cognitive Decline and predict its trajectory

**DOI:** 10.1002/alz70857_103114

**Published:** 2025-12-25

**Authors:** Kevin Duff, Dustin B. Hammers

**Affiliations:** ^1^ Oregon Health & Science University, Portland, OR, USA; ^2^ NIA‐Layton Aging & Alzheimer's Disease Research Center, Portland, OR, USA; ^3^ Indiana University School of Medicine, Indianapolis, IN, USA

## Abstract

**Background:**

Subjective cognitive decline (SCD), defined as cognitive complaints in the absence of objective cognitive impairments, may reflect the earliest manifestation of preclinical Alzheimer's disease (AD). However, individuals with SCD are a heterogenous group with unclear cognitive and clinical trajectories. Building on our prior work examining short‐term practice effects (STPE) in Mild Cognitive Impairment (MCI) and AD, we examined if STPE were diminished in SCD and if they predicted cognitive trajectories.

**Method:**

76 participants classified as cognitively unimpaired according to the Jak/Bondi criteria were divided by whether they presented with cognitive complaints (i.e., SCD, *n* = 11) or not (i.e., CN, *n* = 65). They completed a brief cognitive battery twice across one week (Time 1 to Time 2) to quantify STPE and again after approximately 1.3 years (Time 1 to Time 3) to assess long term cognitive change.

**Result:**

Across one week, those with SCD showed smaller STPE on 7 of the 8 neuropsychological test scores and a composite measure compared to those without cognitive complaints (see Figure 1 for the composite; negative Z‐scores reflect smaller STPE). STPE on visual scanning, set shifting, and processing speed showed the largest effect sizes in separating SCD from CN. In the entire sample, the composite measure of STPE was significantly related to change over 1.3 years (*r* = .31, *p* = .01), with smaller STPE predicting less improvement at long term follow‐up. When considering the two groups, composite STPE and change over 1.3 years showed similar relationships (CN *r* = .23 and SCD *r* = .21). However, when examining processing speed measures specifically, the association between STPE and long‐term changes were stronger in the SCD group (*r* = .91) than the CN group (*r* = .38).

**Conclusion:**

Similar to our work over the past decade in MCI and AD, STPE were reduced in SCD relative to CN, and they predicted cognitive trajectories. As such, these preliminary findings require validation in a larger trial to see if STPE could be used to enrich clinical trials enrolling those with SCD.